# γ-Heregulin has no biological significance in primary breast cancer

**DOI:** 10.1038/sj.bjc.6600245

**Published:** 2002-04-22

**Authors:** E A Sánchez-Valdivieso, J J Cruz, R Salazar, M del Mar Abad, A Gómez-Alonso, A Gómez, R González-Sarmiento

**Affiliations:** Unidad de Medicina Molecular, Universidad de Salamanca, 37007 Salamanca, Spain; Servicio de Oncología, Universidad de Salamanca, 37007 Salamanca, Spain; Departamento de Anatomía Patológica, Universidad de Salamanca, 37007 Salamanca, Spain; Departamento de Cirugía, Universidad de Salamanca, 37007 Salamanca, Spain; Centro de Investigación del Cáncer, Universidad de Salamanca, 37007 Salamanca, Spain

## Abstract

*British Journal of Cancer* (2002) **86**, 1362–1363. DOI: 10.1038/sj/bjc/6600245 www.bjcancer.com

© 2002 Cancer Research UK

## Sir

The short arm of chromosome 8 is one of the most frequently altered in breast cancer. Chromosome 8p abnormalities have been associated with the development and/or progression of breast cancer and 8p22 deletions have been associated with more aggressive phenotypes and poorer survival (Yokota *et al*, 1999; Utada *et al*, 2000). Amplification of the region 8p12, that contains the neuregulin (NRG-1, also denominated heregulin) gene, is detected in about 12% of breast tumours (Adélaïde *et al*, 1994, 1998; Lee and Wood, 1993) and breakpoints in bands 8p11-p21 have been reported in breast cancer (Morris *et al*, 1997; Courtay-Cahen *et al*, 2000). The NRG-1 gene encodes, by alternative splicing or by initiation of gene transcripts at different sites, a family of more than 15 membrane bound or secreted proteins most of which contain an extracellular epidermal growth factor (EGF)-like domain (Peles and Yarden, 1993). These proteins act as ligands for the HER family of receptor tyrosine kinases and play an important role in cell growth and differentiation, morphogenesis and apoptosis (Burden and Yarden, 1997). Although NRG-1 was isolated during the search for a HER-2 ligand (Holmes *et al*, 1992), isoforms of NRG-1 bind to cells that express HER-3 or HER-4 receptors alone, heterodimers of HER-2 with either HER-3 or HER-4 but not to cells that express monomers of HER-2 (Burden and Yarden, 1997).

Much interest in the neuregulins is based on the fact that the receptors for these ligands, the HER family of proteins, are amplified or over-expressed in breast cancer. HER-2 (also known as neu and erbB2) gene amplification and/or over-expression are found in approximately one-third of breast tumours and have been associated with a poor prognosis (Lupu *et al*, 1996). NRG-1 induces an aggressive/invasive transformed morphology in cultured mammary epithelial cells that express HER-2 (Lupu *et al*, 1996) and can stimulate the growth of breast cancer cells that express low levels of the HER-2 receptor (Schaefer *et al*, 1997). Moreover, NRG-1 synthesised by the mesenchyme has been implicated in mammary development (Carraway *et al*, 1997), and it has been demonstrated that NRG-1 induces proliferation or differentiation of various mammary tumour cell lines, initiates programmed cell death and induces cell differentiation in breast tumours (Ram *et al*, 1995; Weinstein *et al*, 1998; Le *et al*, 2000). Recently, it has been identified in MDA-MB-175, a breast carcinoma cell line that shows elevated levels of HER-2 (Lewis *et al*, 1993), a translocation between chromosomes 8p12-21 and 11q13 that leads to the fusion of NRG-1 and DOC4 genes (Schaefer *et al*, 1997; Liu *et al*, 1999; Wang *et al*, 1999; Adélaïde *et al*, 2000). This translocation generates a new chimeric transcript that codes for a new isoform of the neuregulin family, denominated γ-heregulin (γ-HRG), that acts as an autocrine growth factor in the breast cancer cell line MDA-MB-175 (Schaefer *et al*, 1997).

In an attempt to define the incidence of this translocation in Spanish women with breast cancer we have studied this abnormality in a series of 141 breast carcinoma samples collected at the University Hospital of Salamanca. All tumour specimens were frozen immediately after removal and stored at −80°C before processing. DNA was extracted as described (Sánchez *et al*, 1995). The status of γ-HRG gene was assessed by Southern blot analysis. Genomic DNA was digested with the restriction endonuclease *Eco*RI and electrophoresed on 0.8% agarose gels. DNA was blotted and hybridised with a 538 bp *Sac*II-*Xho*I fragment (nt 209–747 from sequence M94165) that spans the putative breakpoint site in chromosome 8p12.

Southern blot analysis of 141 primary breast carcinomas and the MCF-7 breast cancer cell line did not reveal any abnormal fragment. Our results show that the t(8;11)(p12;q13) translocation is not present in a large series of Spanish women with breast cancer and confirm previous reports that suggest that this translocation is a particularity of the MDA-MB-175 cell line and not a recurrent event (Wang *et al*, 1999; Adélaïde *et al*, 2000) indicating that γ-heregulin is not relevant in the development of breast cancer.
